# When *eleven* does not equal *11*: Investigating exactness at a number’s upper bound

**DOI:** 10.1371/journal.pone.0266920

**Published:** 2022-04-28

**Authors:** Ira Noveck, Martial Fogel, Kira Van Voorhees, Giuseppina Turco

**Affiliations:** 1 Université de Paris-Cité, LLF, CNRS, Paris, France; 2 Leiden University, Leiden, Netherlands; Ball State University, UNITED STATES

## Abstract

The *approximate number system* (a) views number as an imprecise signal that (b) functions equivalently regardless of a number’s initial presentation. These features do not readily account for exact readings when a task calls for them. While profiting from insights in areas neighboring the number cognition literature, we propose that linguistic-pragmatic and cultural pressures operate on a number’s upper bound in order to provide exact readings. With respect to (a), Experimental Pragmatic findings indicate that numbers appear to be semantically lower-bounded (*Eleven candidates are coming* means *at least eleven*) but fluid at its upper-bound; *exactly* readings emerge as a consequence of an additional pragmatic process that solidifies the upper bound. With respect to (b), studies from cognitive anthropology underline how symbolic representations of number are distinct from written codes. Here, we investigate a novel hypothesis proposing that symbolic expressions of number (such as “11”) explicitly provide exactly readings unlike verbal (oral and written) ones, which engender *at least* readings. We then employ a Numerical Magnitude Task (NMT), in which French-speaking participants determine whether a presented number is lesser or greater than a benchmark (12) in one of three presentation conditions: i) Symbolic/Hindu-Arabic (e.g. “11” via screen), ii) Oral (e.g. “/ˈon.zə/” via headphones), or; iii) spelled-out-in-Letters (e.g. “onze” via screen). Participants also carry out a Number Identification Task (NIT) so that each participant’s *recognition* speed per number can be removed from their NMT times. We report that decision reaction times to “onze” take longer to process (and prompt more errors) than “treize” whereas “11” and “13” are comparable. One prediction was not supported: Decision times to the critical oral forms (“/ˈon.zə/” and “[tʁ̥ɛːzə̆]”) were comparable, making these outcomes resonate with those in the Symbolic condition.

## 1. Introduction

Investigations into the mental representation of number hold a privileged place in the cognitive sciences. Central to research in this area has been the proposal that we, along with non-human animals, are evolutionarily endowed with an approximate number system (ANS) or the number sense [1]. One of the hallmarks of the ANS is that it accounts for data showing that individuals successfully discriminate between compared quantities as the ratio between them increases, in line with Weber’s law [2, 3]. The bedrock studies that support the ANS have typically been perception experiments in which, for example, participants determine which of two sets of dots are more numerous (e.g. see [4]) or which of two Hindu-Arabic number symbols is greater [5] (for a review, see [6]). A participant who is shown, say, ten yellow dots and five blue dots (a 2:1 ratio of yellow to blue dots) will readily indicate that there are more yellow dots; however, when there are just six yellow dots to five blue ones (reducing the blue:yellow ratio to 1.2:1) the task is more challenging [7] (for critical reviews, see [8, 9]).

Descriptions of ANS usually include three features. One is that number is essentially an imprecise signal, as the word *approximate* suggests. A second concerns modality independence, which highlights the idea that the form of the number expression does not matter to the ANS. That is, number perception at the ANS level should not be dramatically affected when an experiment presents a collection of dots or symbolic Hindu-Arabic numbers. This claim about modalities has led to the *notation-independent hypothesis* in the neuroimagery literature, which assumes that numbers are processed in the same brain region (the Intraparietal Sulcus) whether they are symbolic, written-out in letters, spoken, in Roman numerals, and so on (see [10, 11]). The third feature, *Abstractness*, refers to the notion that performance with numerical materials reflects on “the size of the numbers involved, not on the specific verbal or non-verbal means of denoting them" [12].

Given that it is generally agreed that the ANS is widespread across humans and non-human animals but that only humans can exploit and use exact numbers, a more recent debate concerns the nature of the relation between the ANS and exactness. While there are multiple positions on the human ability to use exact numbers (e.g., see [13–16]; also see [17]), we briefly present two accounts that have animated the debate more recently. One is that meanings for words such as “eleven” emerge directly from the ANS, whether one views the ANS as initially noisy (e.g. see [13, 18]) or precise [19, 20]. This partially explains why work in this area often underlines how individual differences among babies predict performance on Mathematics later [21] or how progressively improving verbal number knowledge can be attributed to increasing ANS acuity among growing 3- to 4-year-olds (e.g., see [22]) and beyond (see [23]). An alternative is that the ANS alone does not provide a direct association for exact number terms; rather, it serves as one of the foundations for eventually establishing exact number abilities [14]. According to this approach, developing children take advantage of three systems to ultimately acquire exact numbers. One system, referred to as parallel individuation, concerns the ability to subitize; this allows children to readily recognize differences between numbers up to three (e.g. [24, 25]). A second is the ANS, which leads one to distinguish among bigger quantities based on ratios [26, 27]. Finally, the third relies on a growing appreciation for the Cardinal Principle along with an eventual competence for counting procedures, both of which put children in a position to learn exact numbers (e.g. see [28]). Although this debate dovetails with the current paper’s subject matter, it was not crucial to the work we carried out. That said, the Discussion will consider how this paper’s outcomes speak to this literature.

The current work aims to contribute to the number cognition literature generally by considering how interpretive factors, stemming from a participant’s effort to determine a presented number’s *meaning*, ultimately affect number processing and comprehension. That is, while we assume that the ANS adequately describes a starting state for number representation, we also contend that when the linguistic-pragmatic framing of a number (the way a number is expressed and the context in which it takes place) is engaged, it prompts processing whose outcomes can appear to eclipse the expectations that are commonly associated with that starting state. For example, while the distance between “eleven” and 12 is equal to the distance between “thirteen” and 12, we expect behavioral outcomes for (lower than/greater than) decisions concerning “eleven” to differ from those concerning “thirteen.” We further assume that these framing considerations are not in play when a participant, for example, is carrying out a perceptual task that involves comparing two images that vary with respect to their number of dots or is estimating the number of items in a stimulus. Our strategy is to consider insights from neighboring cognitive science literatures that investigate number in order to underline how the descriptive power of two ANS features, imprecision and modality independence, retreats somewhat once number meanings in context are taken into account.

In the remainder of the Introduction, we will take the following four steps. First, we review work on the semantics and (experimental) pragmatics of number processing which reveals that, like with non-numerical quantifiers such as *Some* or *Most*, numerical quantifiers come with an *at least* reading (*x objects* means *at least x objects*) that can be informationally enriched by effortfully solidifying the upper bound. The current work essentially aims to determine whether such a finding generalizes to a classic number cognition task. Second, we review work from cognitive anthropology that draws comparisons between symbolic (Hindu-Arabic) expressions of number and written ones (and we view the latter as a proxy for verbal expressions) and thus underlines a distinction between the two. Third, we formulate a hypothesis about the way verbal (oral and written-out expressions of number words), on the one hand, and symbolic (Hindu-Arabic) expressions of number, on the other, differentially lend themselves to exact meanings. We then justify our expectation that symbolic expressions more readily produce exact readings. Finally, we describe an experiment that employs a classic number magnitude task (as inspired by Szucs & Csepé [29]), in which participants determine whether a provided number is greater than or lesser than a benchmark (which in our experiment is the number 12). In this work, we investigate French-speaking participants’ performance across three stimulus modalities (one symbolic, as in “11,” and two that are verbal–one oral, as in “/ˈon.zə/” and another expressed as a written word, as in “onze”).

### 1.1 The semantics and pragmatics of number

Consider (1) below which describes a certain number of mayoral candidates who are joining a parade.

1. Eleven candidates are joining the parade.
2. Eleven and perhaps more candidates are joining the parade (*At least* reading).
3. Eleven candidates and not more than eleven are joining the parade (*Exactly* reading).
4. Some candidates are joining the parade.

Does the number in (1) mean *at least eleven* as in (2) or does it mean *at least and not more than eleven* (viz. *exactly eleven*), as in (3)? According to what can be considered the classical view (e.g. [30]), a number phrase comes lower-bounded, as in (2); however, an exactly reading can emerge pragmatically through an enrichment indicating not more than eleven at its upper-bound (3). This kind of claim about numbers would be consistent with robust findings from the literature on non-numerical quantifiers, such as the one in (4). While the sentence in (4) is semantically compatible with *all candidates are joining the parade*, it plausibly means *Some but not all candidates are joining the parade* (and arguably because the speaker could have used a stronger term, such as *all*, but did not).

### 1.2 What are the empirical grounds for claiming that numbers are not upper-bounded?

We begin by describing data from the studies using non-numerical existential quantifiers, such as *Some*, which show that pragmatic (*Some but not all*) readings of expressions like those in (4) typically come with a cognitive cost. Consider work from Bott & Noveck [31], who reported how false response times to underinformative items, such as “Some cats are mammals,” are particularly long, especially when compared to the speed of true responses. There is a consensus in the Experimental Pragmatic literature that this slowdown is due to a pragmatic enrichment that affects the quantifier’s upper-bound. In other words, an interlocutor can arrive at a solid upper bound (a *Some but not all reading*) by engaging in an effortful pragmatic process (for a view that treats such cases as ambiguous however, see [32]).

This effort-demanding pragmatic effect manifests itself across multiple experimental scenarios, including a) developmental studies showing that children become progressively pragmatic with age (e.g. [33, 34]); b) reading-time studies showing that sentences that contextually call for pragmatic enrichments take longer to process than identical sentences whose contexts do not [35, 36]; c) dual-task studies showing that added cognitive load impairs pragmatic processing among participants [37]; d) visual-world eye-tracking tasks that reveal how participants do not spontaneously seek out instantiations of *not all* readings in the wake of sentences containing *some* [38] and; e) EEG studies showing that pragmatic responding to underinformative sentences (such as *Some pictures contain cats* when they all do) is associated with larger N400’s when compared to control items [39]. For a summary, see [40]. Importantly to our current concerns, scalar implicature studies have been investigated across a wide range of scales (for a relatively complete inventory, see [41]). These include a) modals such as *might*, which is susceptible to a *does not have to* reading [33], b) other existential quantifiers such as *Most*, which leads to enriched readings similar to *Some* [42] and; c) numbers, e.g. how *two* being employed to describe a scenario of three objects leads to developmental effects similar to *Some* [43] (also see [44] for adult priming studies).

Two notable examples come from neuroimagery work on the pragmatic processing of numerical quantifiers. One comes from Spychalska et al. [45], who presented participants with a sentence-picture verification task that begins with a numerically quantified sentence, such as *Three pictures contain*, before showing, say, 3 cats and 5 balls distributed over 6 frames (in a 2 x 3 matrix). To conclude the sentence, a final word was presented (which can be *cats* or *balls* here) and participants were then required to answer with a “yes” or “no.” When the sentence was completed with the word *balls*, the authors reported that two-thirds of participants (30 of 45) rejected the sentence and the remaining third accepted it. These kinds of reaction point to *exact* and *at least* readings, respectively. More importantly, the responses linked to each led to quantitatively different event-related brain potentials (ERPs). For the *exactly* responders the content word (*balls*) prompted a negativity effect relative to a control condition whose concluding noun unproblematically provided an exact reading (such as *cats* here). However, no such effect was evident for the responders who applied an *at least* interpretation (again, these are participants who answer *yes* to *Three pictures contain balls*, when there are five). The other relevant work comes from [46] who presented underinformative quantified sentences to participants in a scanner. It was the participants’ task to say whether there is a match or mismatch when presented a sentence, such as *Three mice have grapes* in the presence of a picture with five mice with grapes. These underinformative sentences typically prompt “mismatch” responses but they are also the source of increased activity in the left anterior middle frontal gyrus (MFG) and medial frontal gyrus (MeFG) compared to controls, which include a condition in which the number in the test sentence overshoots the description, i.e. when the numerical information in the sentence is patently wrong [46]. Taken together, these studies indicate that when a presented number falls just shy of a target, it generates unique activity that can be distinguished from precise uses and even wrong uses. They thus indicate that extra effort (further acuity) is required when the upper bound of a number–as used in a sentence—is forced to come into play. We note here that these investigations concern cases in which the number in a critical test sentence only slightly undershoots the number in a presented scene (i.e. one would not expect such results when, say, “three” is used to describe a scene with dozens of objects).

Incidentally, findings like those in [45] are edifying for the Experimental Pragmatics literature itself, in that they show that enriched readings build on, or are secondary to, *at least* readings. That study arbitrated between several competing accounts of number representation (for a summary of accounts, see [47]). For example, one prominent alternative to the classic account comes from Breheny [48], who proposes an exact semantics for numerical quantifiers from which one can derive a *lower-bounded* reading. A third pragmatic account, from Carston [49], assumes that number is neither lower- or upper-bounded and that context is all determining. Although the experimental pragmatic literature usually mentions these three accounts for numerical terms, investigations usually pit *exact semantic* accounts of number against *classic pragmatic enrichment* accounts, as described above.

### 1.3 Are verbal expressions of number equivalent to symbolic ones?

Cognitive anthropology thoughtfully addresses the mental representation of number as well. Scholars in this area (e.g. [50–52]) describe the cultural evolution of number and the influences behind it. Chrisomalis [50], in particular, convincingly argues that symbolic notations of number are best viewed as cultural inventions that independently evolved for purposes of counting and trade, ultimately making them distinctive from other systematized human expressions, such as written text. Chrisomalis ([50] see page 22) presents four lines of argument in favor of the idea that two systems of human communication–numerals and writing–arose independently. We briefly summarize these here.

First, numerical notations can be used to communicate across cultures, even if their linguistic systems are different. It is telling, Chrisomalis points out, that number systems are more decipherable when embedded with newly discovered ancient notation. Even today, one can imagine the relative efficacy of writing down “11” for the sake of a foreigner to describe the number of articles bought or sold in a market abroad as opposed to writing out “onze” to a non-French-speaker in a similar situation. Second, whereas written and spoken expressions of number overlap structurally (e.g. “thirty” reflects its oral expression /ˈθəːti/) there is not necessarily a one-to-one mapping between numerical notation and verbal ones. For example, while Roman Numerals express thirty as XXX, there are no cases where a language expresses thirty as “10 10 10.” Third, the evolution of numerical and writing systems have different provenances:

The Western numerals diffused initially from India and passed through the Arab world before reaching Europe, while the Roman alphabet is of Greek and Phoenician ancestry. This historical differentiation is not uncommon; the path of diffusion of numerical notation is often radically different from that of the diffusion of scripts.

Finally, tallying systems vary to include knots and notches and often do not make it into written scripts.

This independence between symbolic uses of number that lead to numerical systems such as the Hindu-Arabic one, on the one hand, and scripts expressing verbal (written and oral) expressions of number, on the other, would make it reasonable to assume that the two sets interface with different cognitive processing routes as well. In fact, a perusal of the number cognition literature indicates that symbolic expressions of number often prompt important differences when compared to verbal (i.e. written-out-in-letters) ones. For example, Kadosh and colleagues [53] showed how reaction times vary on a number-comparison task as different features, including notation and the physical size of fonts, are manipulated. In an EEG study, Proverbio et al. [54] showed how Hindu-Arabic symbolic expressions of number (e.g. “28”) generate ERP profiles that differ from “verbal” ones (i.e. written out expressions of number, as in “ventotto” for 28 in Italian) when participants are required to determine whether two numbers (expressed either symbolically or “verbally”) are the same or different.

### 1.4 Proposal: Underdetermined readings for verbal expressions; exact readings for symbolic ones

We begin by advancing the hypothesis that verbally expressed numerical quantifiers operate similarly to non-numerical existential quantifiers, such as *Some*. This would mean that expressions of number are in a position to have a fluid upper bound that can be solidified through a pragmatic enrichment along with an accompanying cognitive cost. However, this claim comes with a caveat. We reserve this to cases where they are expressed verbally or are in a written form that mimics verbal expressions (e.g. the written-out “onze” in French reflects the spoken “/ˈon.zə/”). Essentially, we propose that symbolic, e.g. Hindu-Arabic, representations of number are generally understood with an exact meaning (i.e. they incorporate both a lower- and upper-bound). To put it another way (and less categorically), symbolic expressions of number are more likely than verbal expressions to be understood as exact. Note that our claim is a bit more surgical than those typically found in the number cognition literature, which tend to treat both Hindu-Arabic expressions of number and verbal, written-out-in-letters expressions as symbolic (e.g. see [55], page 8).

Part of our reasoning is inspired by Aronoff’s [56] application of the competitive exclusion principle [57] to the cultural evolution of language, according to which two similar expressions cannot occupy the same niche [56], (also see [58]). While verbal (i.e. oral and written) uses on the one hand and symbolic uses of number on the other co-exist, they are not identical. To the extent that a symbol and a verbal expression share the same niche, one of the two would need to distinguish itself (or become extinct). Part of our proposal is that the symbolic expression of number distinguishes itself by adopting the more precise reading, and verbal expressions a less precise reading, in this shared niche.

In this work, we focus on the two immediate neighbors of a designated number, i.e. the *benchmark*, which sits in the middle of a range of numbers in a numerical magnitude task. If we are on target in assuming that there is a particularized, effort-demanding enrichment that can be specifically extended to a verbally expressed number’s upper-bound, it implies that the decision (to indicate whether a presented number is lesser or greater than the benchmark) about the number immediately *below* the benchmark ought to provide slower reaction times (and increased error rates) when compared to the decision about the number immediately *above* it. That is because the number immediately below the benchmark requires extra (pragmatic) processing to distinguish it from the benchmark, unlike the number above it (that number’s meaning has an *at least* reading and does not need precision at its upper bound in order for the participant to answer). This is the kind of scenario found in Numerical Magnitude Tasks (NMTs), such as the one employed by Szucs & Csepé [29] that serves as our inspiration. In their task, Hungarian participants were presented sixty trials of each of the numbers 1 through 4 and 6 through 9 (either orally or symbolically) as they decided whether the presented stimulus number is inferior, or superior, to 5. Though these authors did not investigate the difference between the oral presentations for 4 (*négy* in Hungarian) and 6 (*hat*) one can see that there is an uptick in errors and reaction times for the former (see Table 1 in Szucs & Csepé, 2005, which is also made available in our pre-registration). Other data in line with this hypothesis can be found in the remaining experiments from their paper as well as from other articles in the literature. For example, Dehaene [10] used a version of the NMT and reported an interaction indicating that the spelled out *four* (in English) took longer to answer with a “smaller (than)” button-press than a *six* did for a “larger (than)” button press. At the very least, one can see that there is evidence in the literature to support our hypothesis that verbal expressions of number immediately below a benchmark prompt extra processing when compared to the number immediately above it, which serves as a control. These sorts of findings are rarely pursued because the working assumption in the number cognition literature is that the distance from the benchmark is critical, regardless of the direction between the stimulus number and the benchmark (e.g. see [5]).

## 2. Experiment

We carried out an NMT modeled on Szucs & Csepé ([29], Experiment 1) while making three critical modifications that turn the experiment into a more complete and stringent test of our claims. First, while we began our investigation focused on symbolic Hindu-Arabic numbers and orally-presented expressions of number, as Szucs & Csepé did, we extended our modes of presentation to numbers written-out-in-letters because, as described above, there is prior work indicating that lexical presentations of number appear to differ from symbolic ones. Second, given the testing language (French), we moved the range of numbers away from 1–9 in order to avoid (a) phonological ambiguities linked to critical spoken words (i.e. the initial voicing of *six* in French overlaps phonologically with *sept*), and, more generally, to avoid; (b) numbers in the subitizing range. The adjustment in (b) is especially important because nearly all NMT’s that use 1 through 9 (with 5 as a benchmark) necessarily include an important subset containing subitizing numbers; this means a majority of numbers below the benchmark can appear exceptionally fast but not necessarily because of their distance from 5 but because they are quickly identified when subitized (making the comparison to 5 easier downstream). We elected to use the number range 8–16 (with 12 as the benchmark). This way all the numbers are far from the subitizing range of numbers and the two numbers that are to be critically compared in this set–“onze” and “treize”–are phonologically unique in the experiment. Note too that “onze” in terms of its length is shorter than “treize”; assuming that word length affects processing (see [10]), the intended comparison works against our hypothesis because we predict the former (shorter) number to take longer to process than the latter. In a similar vein, we point out that while “onze” and “treize” are frequently used in French according to the Lexique database [59], “onze” is more frequent than “treize” (in every media source including news, film and twitter), which should make its processing faster. Finally, we created a follow-up Number Identification Task (NIT), under conditions similar to the NMT but without the *inferior/superior* task. This provides mean identity times—for each number and per participant–that can be subtracted essentially from their RT’s on the NMT, resulting in a cleaner decision time measure.

We note here that our OSF registration, https://osf.io/kz7b6/, was focused chiefly on our lengthy efforts to transform Szucs & Csepé’s [29] Numerical Magnitude Task into French as we developed the Oral condition. Afterward, we succinctly added that we will also include a second “experiment” involving visual symbolic numbers. Although we intended from the start to include all three conditions that are in the current work (what we call the *Oral*, *Symbolic*, and *Letters* conditions), the pre-registration will appear incomplete. Our registration’s apparent shortsightedness (to not include the *Letters* condition) is due to the fact that it was prepared partly for pedagogical purposes (for one co-author’s Masters) and we anticipated having enough time to carry out the first two conditions only (which were carried out serially). The Letters condition was carried out as another iteration of the same class of “Experiment” immediately after the Masters was submitted. As will become clear below, we present the three “Experiments” as different conditions under one *modality* umbrella.

### 2.1 Method

#### 2.1.1 Participants

Thirty-six native French speakers participated in the study. All were native French speakers from Lyon, who were recruited through local advertisements that targeted students from the local Universities and who were offered a gift worth approximately €10 for participation. The experiment received approval from a National French Ethics committee, known as Comité de Protection des Personnes (CPP), Sud-Méditerranée I (2019-A00681-56).

#### 2.1.2 Materials and procedure

Each number was randomly presented 60 times, which makes for 480 trials. Oral numbers were provided over headphones whereas the Hindu-Arabic symbols and those presented as written words were presented on a computer screen. Instructions described the task and explained that participants should hit one key if the presented number is *inferieur à 12* and the other if it was *superieur à 12*. Before each trial, a fixation point in the form of a cross appeared for one second to help prepare the participant for the arrival of the stimulus. The number–whether it was in the Symbol, Oral or Letters condition–was part of a two-second presentation. Thus, each trial lasted three seconds. Participants were asked to answer promptly.

Note that when we refer to the condition name with respect to number we will present it in capitals, as in Number. When we refer to some category of Number (any one of the eight stimuli ACROSS modalities), i.e. its numerical value, we will refer to it as a bare number (as in 8). In contrast, when we refer to a number in a specific modality, we will distinguish it by putting it in quotes and by designating it symbolically, lexically or phonetically, as in “8,” “huit” or “*/ɥit/*”, respectively.

The trials were presented in two blocks, each containing 240 trials. For one block, participants were asked to press the D key (on an AZERTY keyboard) for *inferieur à* 12 and the L key for *superieur à* 12. For the other block, the meaning of the keys was reversed. The presentation of these blocks was randomized. Before each block, there were 72 training trials with feedback; a buzzer sound would indicate an error and a chime would indicate a correct response. The training encouraged rapid responses. Responses taking longer than 2 seconds were accompanied by the buzzer. Participants were allowed to take a break between the two blocks.

**Fig 1:**
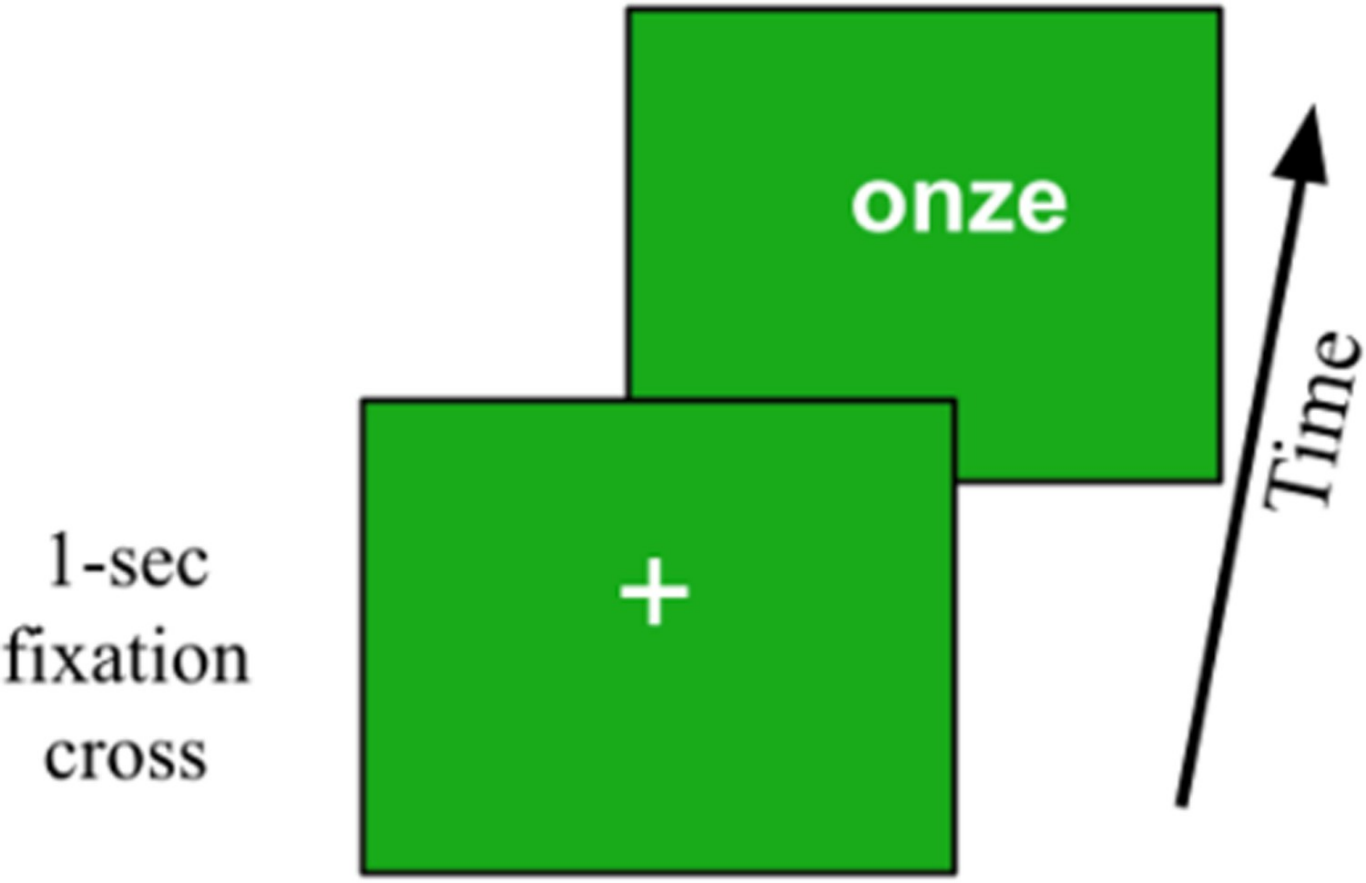
An example item from the Letters condition of the Numerical Magnitude Test (the NMT). Participants have up to two seconds to indicate whether the number is less than or greater than the benchmark (by responding with “D” or “L”) from the moment the number appeared.

All participants were seated in front of a portable computer in a quiet lab room and they responded through its keyboard. The *Oral* group received the stimuli, prepared by a native speaker of French in a phonology lab in Paris, through headphones (thus eleven-in-French here was expressed as “/ˈon.zə/”). The *Symbol* and *Letters* groups received numbers on its screen, symbolically (e.g. as “11”) and as written-letters (e.g. as “onze”), respectively. Safeguards were included to avoid consecutive identical stimuli as well as three consecutive uses of the same response key (whether it be for *inferieur* or *superieur*). See Fig 1 for an example presentation of the NMT.

**Fig 2:**
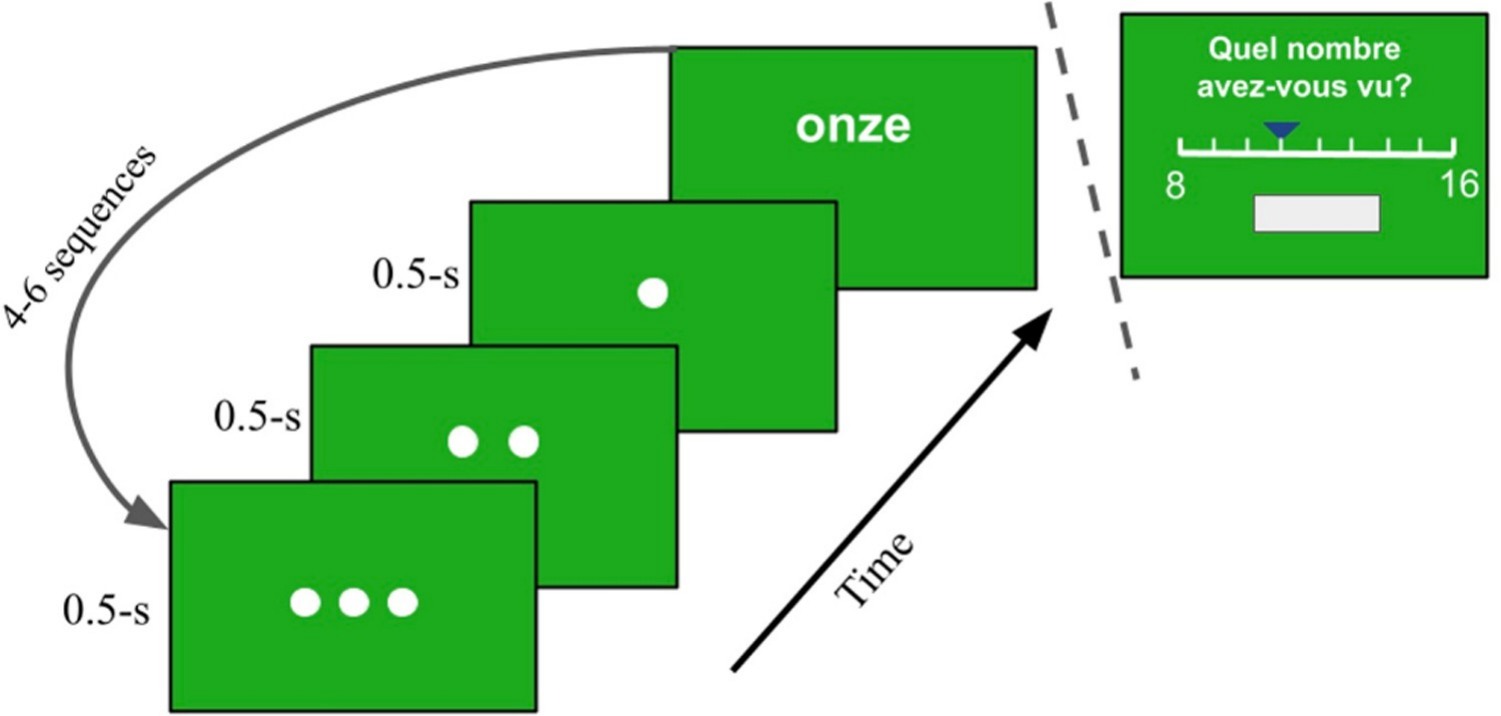
Number Identification Task. Post countdown, participants view a number (in their condition’s modality) and tap the spacebar as soon as they identify it. On occasion (every 4 to 6 sequences), participants are asked to produce the number last seen (or heard). The box below the scale (see upper right) shows the number on the scale (in symbolic form) as the participant moves the cursor horizontally.

The follow-up NIT presented participants with a number (in the same form as the NMT) while asking for an identification as-quickly-as-possible (by tapping the space bar). A three-step signal (a racecar style colored countdown over one and a half seconds) prepared participants for the number’s arrival. Every four to six trials, participants were asked to specify the last number heard (or seen) through an on-screen scale (ranging from 8 to 16, *sans* 12). A scaling trial would conclude when the participant clicked on the box below the scale (see Fig 2), which contained the number (shown in symbolic form) the participant identified. The NIT task ended when each number was *scaled* 4 times. Both tasks were prepared with *Psychopy* software [60]. The task’s duration was on average 35 minutes.

#### 2.1.3 Results

**Fig 3:**
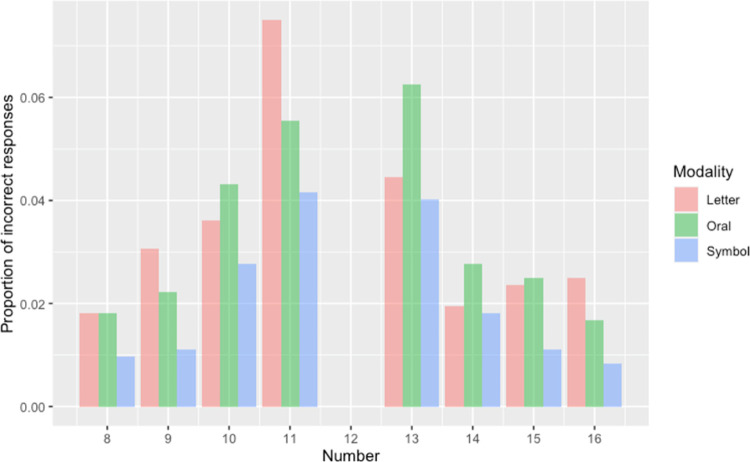
Error rates on the superior/inferior decision for the NMT across three modalities and range of numbers.

We are concerned with two Dependent variables–Accuracy and Reaction Times–and begin with the former. The accuracy data–determined by participants’ responses to the *inferieur*/*superieur* question—stem from the 36 participants (twelve participants across three modality conditions, which will be referred to as *Oral*, *Symbol*, and *Letters*) and the 5760 data points that each condition generates (making for a total of 17280). Accuracy was quite high, 97.03% overall. We tested whether Accuracy was significantly different across the provided eight numbers and across modalities while using a mixed logistic regression model (see, for instance, [61]) that included two fixed factors, Number and Modality (see Fig 3).

Analyses were carried out with R software [62] using the *glmer*-() function from the *lme4* package [63]. The model revealed no interaction and no main effect of Modality. However, there was an effect of Number for the following three classes of items: 10 (β: -0.91, SE = 0.33, z = -2.75, p< .01), 11 (β: -1.19, SE = 0.32, z = -3.70, p< .001) and 13 (β: -1.32, SE = 0.31, z = -4.16, p< .0001). See Fig 3. This indicates that these three numbers led to fewer correct responses (or, to put it another way, to higher error rates) with respect to the number 8 (which represents the Intercept of the model for the factor Number). More specifically, since the estimate for 10 is -0.91 and 1—e^-0.91^ = 0.60, this means that the odds that 10 leads to an error increases by 60% when compared to the error rate of 8. Likewise, since the estimate for 11 is -1.19 and 1—e^-1.19^ = 0.70, this means that the odds of 11 leading to an error increases by 70% when compared to the error rate of 8. Finally, since the estimate for 13 is -1.32 and 1—e^-1.32^ = 0.73, this means that the odds of 13 leading to an error increases by 73% with respect to the error rate of 8.

It is more germane to our investigation to determine whether expressions of 11 prompt more errors than 13 and so we carried out the same type of model as above with responses to “/ˈon.zə/” (the Oral modality of the 11 condition) serving as the reference. With 4320 observations across the three modality conditions, we found an interaction (β_13*Lett_: -0.70, SE = 0.32, z = -2.18, p < .05). We were thus motivated to isolate the effects behind this interaction and thus compared 11 to 13 in each of the modalities. We found an effect within the Letters modality, i.e. for “onze” versus “treize” (β: -0.57, SE = 0.23, z = -2.50, p< .05). As Fig 3 shows, “onze” (the *Letters* modality for 11) prompts higher error rates than “treize” (the *Letters* modality for 13). We found no such effects among the numbers in the Oral modality; in fact, “/ˈon.zə/” prompts slightly fewer errors than “/tʁɛz/”. Likewise, “11” and “13” appear to prompt equivalent rates of errors. Overall, performance with the two numbers in the Symbolic condition tend to be less prone to error than those in the other modalities.

**Fig 4:**
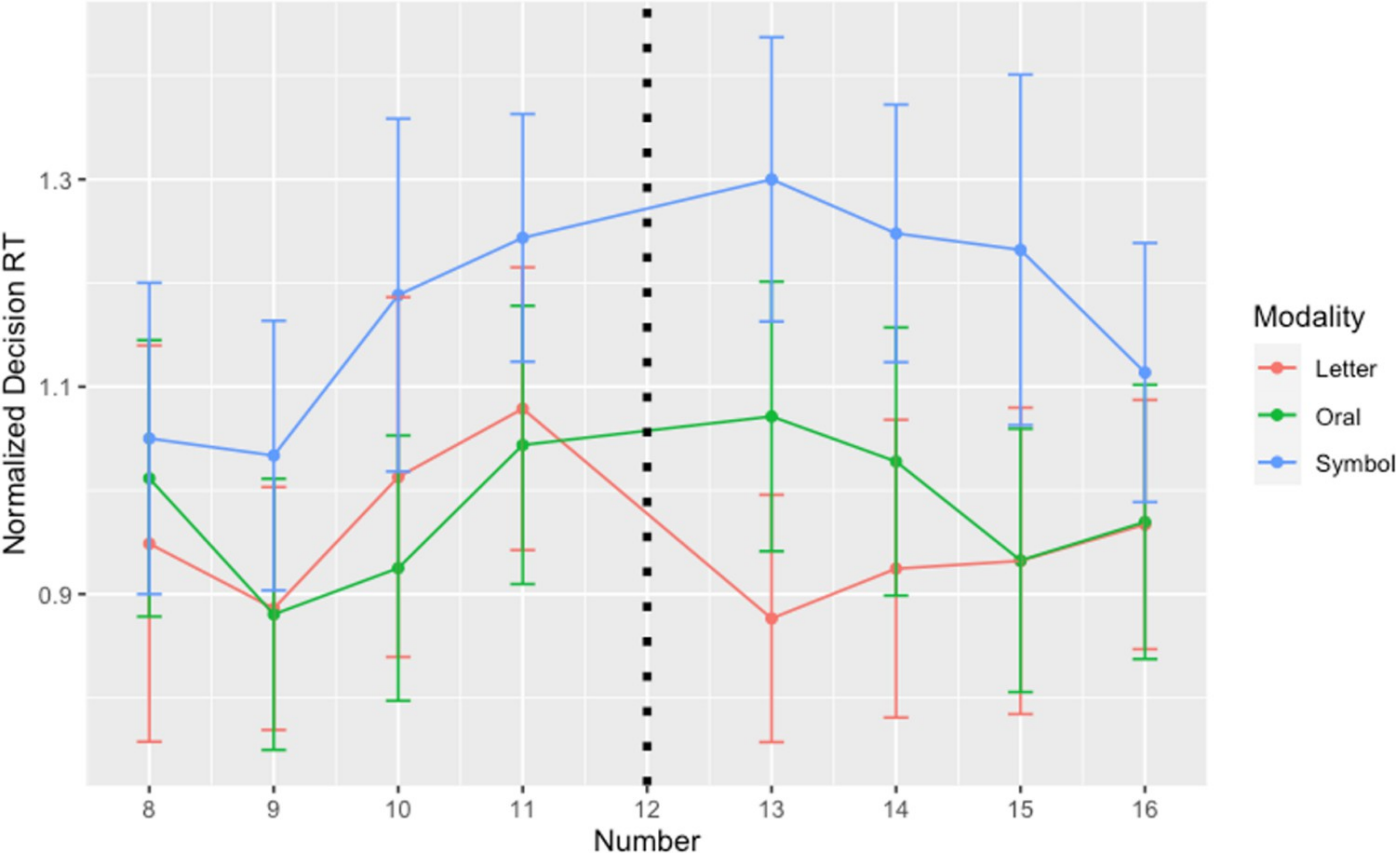
Normalized decision RT’s by Number and Modality. Each data point represents the mean RT across participants (for each modality) after removing each participant’s NIT reaction time per Number from each relevant NMT reaction time, calculated as log(RT_NMT_/ RT_NIT_ by-participant mean per number). Whiskers denote standard error.

We now turn to the critical reaction time data. We will begin by analyzing the results across all the stimuli in the Number condition and then focus on the RT’s concerning 11 vs. 13 across the three modalities. It is important to describe the three steps we took in order to be in a position to analyze our RT data. First, for each participant, we kept only correct responses. Second, we removed data points that were 2.5 standard deviations above or below the participant’s mean (this amounted to 2.8% of the data) by using the *LMERConvenienceFunctions* package [64]. Finally, to arrive at a cleaner measure of decision reaction times while also normalizing the data to adjust for the positive skew of the NMT’s RT, we performed a log-transformation on each RT on the NMT divided by each participant’s mean RT per Number on the NIT. This amounts to the following formula: log(RT_NMT_/ RT_NIT_ by-participant-mean per number). We will refer to this as the Normalized Decision Reaction Time (or NDRT). In this way the NIT speed per participant and per number is removed in a principled way from each reaction time on the NMT. A summary of the overall results with this calculated mean can be seen in Fig 4. To better appreciate the two sources for the NDRT, we include Tables 1 and 2, which report the mean raw Reaction Times for the NMT across conditions as well as the mean raw Reaction Times *across* participants on the NIT.

**Table 1:** Mean RT (in ms) on the Number Magnitude Task (NMT), by Number and Modality.

				Number				
Modality	8	9	10	11	13	14	15	16
**Letters**	572	587	600	635	609	577	604	584
**Oral**	920	967	984	967	994	980	885	979
**Symbol**	580	573	634	672	699	629	607	594

**Table 2:** Mean RT (in ms) on the Number Identity Task (NIT), by Number and Modality.

				Number				
Modality	8	9	10	11	13	14	15	16
**Letters**	288	239	243	228	261	269	261	248
**Oral**	325	390	379	351	344	347	374	367
**Symbol**	223	252	203	197	216	181	219	208

Note. These are global means and thus do not reflect how each participant’s mean per number served as a denominator to calculate the Normalized Decision Reaction Times.

To investigate the two conditions, we performed likelihood ratio tests using the *anova* function [65] and a linear-mixed effects model testing the relation between the NDRT and the following fixed factors: Number (8 through 16, *sans* 12), Modality (Oral, Symbol, Letter,), and Key-orientation (DL, LD). We found a main effect of Number (χ^2^(42) = 1166.3, p < .0001), a main effect of Modality (χ^2^(32) = 559.95, p < .0001), and an interaction between Number and Modality (χ^2^(28) = 550.33, p < .0001). All the other interactions–between a) Key-orientation and Modality; b) Key-orientation and Number, and; c) Key-orientation, Number and Modality—were not significant (all *p*-values > 0.5). Key-orientation was not of theoretical relevance in any case and we do not (and need not) consider it further.

**Fig 5:**
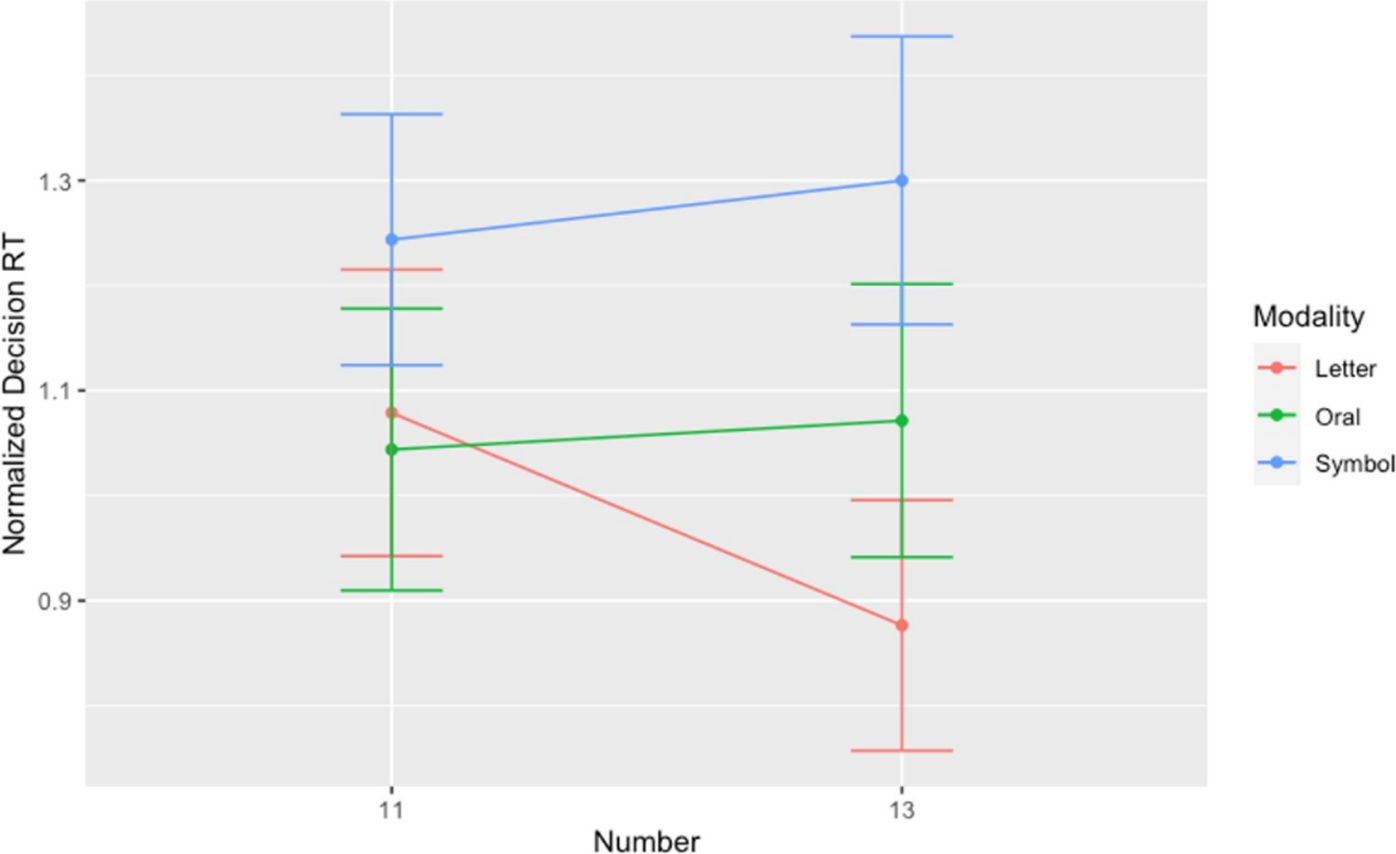
A magnified view on the Normalized Decision Reaction Times for the numbers 11 and 13 across the Experiment’s three modalities. Whiskers denote standard error.

Post-hoc analyses (by the *emmeans* package [66]) were performed on the benchmark 12’s immediate neighbors via pairwise comparisons on the estimated means within each modality (see Fig 5). In line with our hypothesis, we found that within the Letters modality, “onze” led to slower NDRT’s when compared to “treize” (β = .198; SE = .015, z-ratio = 13.21, p < .0001; for the other modalities, the p-values were greater than 0.1). Thus, we do not report a finding indicating that an Oral presentation prompts prolonged reaction times with respect to the number immediately below the benchmark, as we had predicted and as S&C’s (2005) data intimated, but we do find evidence confirming our prediction with respect to the Letters modality condition.

Following up on a suggestion from a Reviewer, we extended our post-hoc comparisons among the pairs of 12’s neighbors in order to assuage potential doubts that the above effect is perhaps local or due to uncontrollable and unforeseen factors. We thus expanded outward to the number expressions “dix” (10 in the Letters modality) and “quatorze” (14 in the Letters modality) and, indeed, found slower NDRT’s for the former compared to the latter (β = .079; SE = .015, z-ratio = 5.43, p < .0001). This was not a planned comparison but it is in line with our hypothesis and it is consistent with our earlier finding indicating that 10 (along with 11 and 13) prompts exceptionally high error rates. No other comparison of this sort revealed a significantly slower NDRT for an “inferior” response when compared to its “superior” cohort and across the three modalities.

## 3. Discussion

This study investigated comparisons on a particularized number line as we focused on comparing a pair of numbers, i.e. the NDRT’s of 11 and of 13 with respect to 12, the benchmark, across three modalities. The study was inspired by two related results in the Experimental Pragmatic literature. One is a robust finding showing that existential quantifiers, such as *Some*, are readily interpreted with an *at least* reading and are further enriched pragmatically with cognitive effort at its upper bound (to mean *and not all*). The other is that this kind of result has been extended to numerical quantifiers (Spychalska et al., 2019) all the while being consistent with other results (Shetreet et al., 2014). In light of such findings, effects in number cognition tasks appeared sensible too; for example, the finding that “four,” when immediately below an NMT’s benchmark, prompted longer RT’s than “six” (as documented in Dehaene [1996]) fit with those in the experimental pragmatic studies. We further refined our predictions in light of work from cognitive anthropologists (especially Chrisomalis, 2010), which underlines a distinction between symbolic and written expressions of number. This led us to investigate performance on the Numerical Magnitude Task (with performance normalized relative to our Number Identity Task) while making the prediction that a) verbal–written-out-in-letters and oral—expressions of number call for pragmatic enrichment at a presented number’s upper bound but that; b) presented symbolic expressions *incorporate* solidified upper-bounded readings.

We confirmed our prediction concerning the critical numbers in the Letters condition, i.e. “onze” (11 in the Letters modality) generates longer NDRT’s as well as more errors than “treize” (13 in the Letters modality). This is an important finding because most researchers assume that the neighboring (equidistant) numbers below and above a benchmark generally behave equivalently in numerical magnitude tasks, starting with Moyer & Landauer (1967). Our reported finding is intriguing because the paradigm was set up to severely test our hypothesis. The version of the NMT we put in place includes a) a range of numbers that are novel to this literature and well beyond the subitizing range, b) a comparison of numbers that end up having much smaller ratios than is typically found in NMT studies and; c) an expression of *eleven* in French that is shorter than its cohort for *thirteen*. We also confirmed our prediction with respect to the Symbol condition in that there were no apparent differences between “11” and “13” (this might seem unsurprising since this outcome is consistent with assumptions in the number cognition literature).

One prediction—concerning the Oral condition—was not supported. We found no slowdowns (or increases in error rates) for “/ˈon.zə/” when compared to “/tʁɛz/.” We thus ask, why would the Oral condition appear to differ from the Letters condition and why would the Oral condition produce a null effect? We have three hypotheses that address these questions. One is based on the observation that the raw reaction times (in both the NMT and NIT) in the Oral condition are generally longer as can be seen in Tables 1 and 2. That is, while the NDRT’s for the Oral condition are generally comparable to those in the Letters conditions (as seen in Fig 4), their globally slow uptakes (both to make a decision about orally presented numbers and to quickly identify them) allow the listener more time to enrich the meaning of orally presented numbers in parallel, even if this effort is gratuitous for numbers above 12. An alternative to the first hypothesis is that the pragmatic slowdown effect that is visible with “onze” in the Letters condition manifests itself *because* written number words, in particular, can be processed quickly and that it is this speed-up that exposes a minimal semantic (*at least*) meaning. Note that this hypothesis runs opposite to the first one. That is, according to this alternative, there are cultural pressures that have been exerted on number comprehension that arguably operate, not over the symbolic representations that encourage exact readings but, over the written codes that allow addressees to process information in a way that is ultimately speedier and weaker than oral expressions. A third hypothesis is that orally presented numbers for this task simply access the same sources as the Symbolic representations. One could argue that the benchmark and the number line are directly accessible in the Oral modality (one need not *read* anything before embarking on number comparisons). Further research could better determine which of these hypotheses is best supported.

Here, we turn to another observation that became evident over the course of the investigation. That is, the effects related to “onze” in the Letters condition can be extended to “dix” (10 in the Letters modality). As can be seen in Fig 4 and then confirmed statistically in the Results section, the rounded, more frequent and shorter-in-length number “dix” prompts slower NDRT’s than its eight-letter-long cohort “quatorze” (14 in the Letters condition). We did not anticipate making this comparison so we did not initially pursue this contrast, but we also find it telling that 10, unlike 14, was associated with higher error rates. One could skeptically conclude that such findings only add to the corpus of data on magnitude and naming tasks across modalities which tend to show mixed results (e.g., see [67, 68]). However, the current work’s critical NDRT subtracts identity times away from magnitude comparison judgments, two response times that likely differ based on modality and task (see Tables 1 and 2). Arguably, our NDRT measure provides a modicum of precision that has not been employed in the literature until now and could be important for future tests investigating number acuity.

Our reported findings can potentially impact the scalar implicature literature as well. For one thing, they bolster claims from Spychalska et al. [45] who showed that underinformative uses of numerical quantifiers, such as “three cats” used to describe a situation that contains five, prompt outcomes that are consistent with those found when existential quantifiers, such as *some*, are used underinformatively to describe a scenario depicting a situation showing *All* [39]. Note, too, that Spychalska et al. [45] used number words written out in letters. The current findings also highlight what distinguishes Marty et al. [69] from other studies in the literature. Marty et al. [69], who requested metalinguistic acceptability ratings of oftentimes weakly worded quantified statements from participants while under one of two working memory loads, directly compared performance between underinformative uses of *Some* (*Some dots are red* when all were red) and underinformative uses of *4* (*4 dots are red* when seven were). They reported an intriguing interaction, in which the underinformative sentences with *Some* were rated more highly (i.e. as closer to being a correct depiction or *True*) under the heavy working memory load than the lighter one, which is consistent with earlier findings indicating that scalar implicature is less likely to be carried out when participants’ cognitive resources are taxed (e.g. see [37]), thus increasing the chances that participants will process weakly worded sentences with a semantic (*Some and perhaps all*) reading. This much is not surprising. What is surprising is that participants in Marty et al. [69] gave *higher* ratings of *True*-ness when presented a sentence like *4 dots are red* when there were seven and when they were operating under a relatively light cognitive load as opposed to under a relatively heavy cognitive load. This indicates that participants are more likely to employ exact meanings when they are burdened with a heavier cognitive load; in contrast, they are more likely to in effect *undo* an exact reading (i.e. by generating an *at least* reading when they are operating under a lighter load. As these examples make clear, Marty et al. [69] used bare numerals (a Symbolic notation) in their test sentences when they reported this effect plus the ratio between the number in the test sentence and the one in the test-picture was consistently high, 1.75:1 (i.e. there were no cases in which participants were asked to make judgments about neighboring numbers such as “6” when there were seven dots). Both are features that arguably encourage an *exact* reading and would explain why their data provide results that go counter to what one typically finds in the Experimental Pragmatic literature.

Here, we consider how our data informs the emergence-of-exactness debate in the number cognition literature. It strikes us that the asymmetric imprecision that is apparent in the Letters condition—when fine comparisons are called for—indicates that deriving exact readings from everyday expressions of number remains slightly unstable even among educated adults and that exactness depends on the context in which the number is used, i.e., the location of an upper limit for the purposes of a task will be critical along with the form in which that number is expressed. These data show that arriving at a precise meaning includes extra effort that involves refining a number’s upper bound. While it is hard to deny the notion that the ANS provides a starting state, it also appears that linguistic and cultural pressures encourage us individually and collectively in an almost whiggish way to adopt exact representations of number.

To conclude, we have shown that bringing together concepts and techniques from areas that neighbor the number cognition literature can be edifying to all. We have provided evidence showing that precision is sought at a number’s upper bound as participants seek to decipher a number’s meaning. We have also shown that the modality in which a number is presented is likely to play a role in the emergence of exact readings.

## References

[1] Dehaene S. The number sense. New York: OUP; 1997. 352 p.

[2] Dehaene S, Piazza M, Pinel P, Cohen L. Three parietal circuits for number processing. Cognitive neuropsychology. 2003 May 1;20(3–6):487–506. doi: 10.1080/02643290244000239 20957581

[3] Feigenson L, Dehaene S, Spelke E. Core systems of number. Trends in cognitive sciences. 2004 Jul 1;8(7):307–14. doi: 10.1016/j.tics.2004.05.002 15242690

[4] Barth H, Kanwisher N, Spelke E. The construction of large number representations in adults. Cognition. 2003 Jan 1;86(3):201–21. doi: 10.1016/s0010-0277(02)00178-6 12485738

[5] Moyer RS, Landauer TK. Time required for judgements of numerical inequality. Nature. 1967 Sep;215(5109):1519–20. doi: 10.1038/2151519a0 6052760

[6] Cantlon JF, Platt ML, Brannon EM. Beyond the number domain. Trends in cognitive sciences. 2009 Feb 1;13(2):83–91. doi: 10.1016/j.tics.2008.11.007 19131268 PMC2709421

[7] Libertus ME, Odic D, Feigenson L, Halberda J. Effects of visual training of approximate number sense on auditory number sense and school math ability. Frontiers in psychology. 2020:2085. doi: 10.3389/fpsyg.2020.02085 32973627 PMC7481447

[8] Guillaume M, Van Rinsveld A. Comparing numerical comparison tasks: a meta-analysis of the variability of the Weber Fraction relative to the generation algorithm. Frontiers in Psychology. 2018:1694. 10.3389/fpsyg.2018.01694 PMC6142874 30271363

[9] Price GR, Palmer D, Battista C, Ansari D. Nonsymbolic numerical magnitude comparison: Reliability and validity of different task variants and outcome measures, and their relationship to arithmetic achievement in adults. Acta psychologica. 2012 May 1;140(1):50–7. doi: 10.1016/j.actpsy.2012.02.008 22445770

[10] Dehaene S. The organization of brain activations in number comparison: Event-related potentials and the additive-factors method. J Cogn Neurosci. Winter 1996; 8(1): 47–68. doi: 10.1162/jocn.1996.8.1.47 23972235

[11] Pinel P, Dehaene S, Riviere D, LeBihan D. Modulation of parietal activation by semantic distance in a number comparison task. Neuroimage. 2001 Nov 1;14(5):1013–26. doi: 10.1006/nimg.2001.0913 11697933

[12] Dehaene S, Dehaene-Lambertz G, Cohen L. Abstract representations of numbers in the animal and human brain. Trends in neurosciences. 1998 Aug 1;21(8):355–61. doi: 10.1016/s0166-2236(98)01263-6 9720604

[13] Dehaene S. The case for a notation-independent representation of number. Behavioral and brain sciences. 2009 Aug;32(3–4):333–5.

[14] Carey S, Barner D. Ontogenetic origins of human integer representations. Trends in cognitive sciences. 2019 Oct 1;23(10):823–35. doi: 10.1016/j.tics.2019.07.004 31439418

[15] Gelman R, Gallistel CR. Language and the origin of numerical concepts. Science. 2004 Oct 15;306(5695):441–3. doi: 10.1126/science.1105144 15486289

[16] Frank MC, Everett DL, Fedorenko E, Gibson E. Number as a cognitive technology: Evidence from Pirahã language and cognition. Cognition. 2008 Sep 1;108(3):819–24. doi: 10.1016/j.cognition.2008.04.007 18547557

[17] Clarke S, Beck J. The number sense represents (rational) numbers. Behavioral and Brain Sciences. 2021;44. 10.1017/S0140525X21000571 33843510

[18] Piazza M. Neurocognitive start-up tools for symbolic number representations. Trends in Cognitive Sciences. 2010 Dec 1;14(12):542–51. doi: 10.1016/j.tics.2010.09.008 21055996

[19] Halberda J. Epistemic limitations and precise estimates in analog magnitude representation. In: Barner D & Baron AS editors Core knowledge and conceptual change. 2016 Jun 30:171–90.

[20] Gallistel CR. The approximate number system represents magnitude and precision. Behavioral and Brain Sciences. 2021;44. doi: 10.1017/S0140525X21000959 34907865

[21] Libertus ME, Feigenson L, Halberda J. Preschool acuity of the approximate number system correlates with school math ability. Developmental science. 2011 Nov;14(6):1292–300. doi: 10.1111/j.1467-7687.2011.01080.x 22010889 PMC3338171

[22] Shusterman A, Slusser E, Halberda J, Odic D. Acquisition of the cardinal principle coincides with improvement in approximate number system acuity in preschoolers. PloS one. 2016 Apr 14;11(4):e0153072. doi: 10.1371/journal.pone.0153072 27078257 PMC4831828

[23] Halberda J, Feigenson L. Developmental change in the acuity of the" Number Sense": The Approximate Number System in 3-, 4-, 5-, and 6-year-olds and adults. Developmental psychology. 2008 Sep;44(5):1457. doi: 10.1037/a0012682 18793076

[24] Feigenson L, Carey S. Tracking individuals via object‐files: evidence from infants’ manual search. Developmental Science. 2003 Nov;6(5):568–84.

[25] Feigenson L, Carey S. On the limits of infants’ quantification of small object arrays. Cognition. 2005 Oct 1;97(3):295–313. doi: 10.1016/j.cognition.2004.09.010 16260263

[26] Spelke ES. Core knowledge. American psychologist. 2000 Nov;55(11):1233. doi: 10.1037//0003-066x.55.11.1233 11280937

[27] Xu F, Spelke ES. Large number discrimination in 6-month-old infants. Cognition. 2000 Jan 10;74(1):B1–1. doi: 10.1016/s0010-0277(99)00066-9 10594312

[28] Sarnecka BW, Wright CE. The idea of an exact number: Children’s understanding of cardinality and equinumerosity. Cognitive science. 2013 Nov;37(8):1493–506. doi: 10.1111/cogs.12043 23672476 PMC3830647

[29] Szűcs D, Csépe V. The parietal distance effect appears in both the congenitally blind and matched sighted controls in an acoustic number comparison task. Neurosci Lett. 2005; 384(1–2): 11–16. doi: 10.1016/j.neulet.2005.04.050 15893429

[30] Horn L. A natural history of negation. University of Chicago Press; 1989. 694 p.

[31] Bott L, Noveck IA. Some utterances are underinformative: The onset and time course of scalar inferences. Journal of Memory and Language. 2004 Oct 1;51(3):437–57.

[32] Chierchia G, Fox D, Spector B. The grammatical view of scalar implicatures and the relationship between semantics and pragmatics. Semantics: An international handbook of natural language meaning. 2012;3:2297–332.

[33] Noveck IA. When children are more logical than adults: Experimental investigations of scalar implicature. Cognition. 2001; 78(2): 165–188. doi: 10.1016/s0010-0277(00)00114-1 11074249

[34] Pouscoulous N, Noveck IA, Politzer G, Bastide A. (2007). A developmental investigation of processing costs in implicature production. *Language Acquisition* 14(4): 347–375. 10.1080/10489220701600457

[35] Breheny R, Katsos N, Williams J. Are generalised scalar implicatures generated by default? An on-line investigation into the role of context in generating pragmatic inferences. Cognition. 2006; 100(3): 434–463. doi: 10.1016/j.cognition.2005.07.003 16115617

[36] Bergen L, Grodner DJ. Speaker knowledge influences the comprehension of pragmatic inferences. J. Exp. Psychol.: Learn Mem Cogn. 2012; 38(5), 1450. doi: 10.1037/a0027850 22545611

[37] De Neys W, Schaeken W. When people are more logical under cognitive load: Dual task impact on scalar implicature. Exp psychol. 2007; 54(2): 128–133. doi: 10.1027/1618-3169.54.2.128 17472096

[38] Huang Y T, Snedeker J. Online interpretation of scalar quantifiers: Insight into the semantics–pragmatics interface. Cogn psychol. 2009; 58(3): 376–415. doi: 10.1016/j.cogpsych.2008.09.001 18976987

[39] Spychalska M, Kontinen J, Werning M. Investigating scalar implicatures in a truth-value judgement task: Evidence from event-related brain potentials. Lang Cogn Neurosci. 2016; 31(6): 817–840. 10.1080/23273798.2016.1161806

[40] Noveck IA. *Experimental pragmatics*: *The making of a cognitive science*. Cambridge University Press; 2018. doi: 10.1075/pc.19023

[41] Van Tiel B, Van Miltenburg E, Zevakhina N, Geurts B. Scalar diversity. Journal of semantics. 2016 Feb 1;33(1):137–75.

[42] Ariel M. Most. Language. 2004;80(4):658–706.

[43] Papafragou A, Musolino J. Scalar implicatures: experiments at the semantics–pragmatics interface. Cognition. 2003; 86(3): 253–282. doi: 10.1016/s0010-0277(02)00179-8 12485740

[44] Rees A, Bott L. The role of alternative salience in the derivation of scalar implicatures. *Cognition*. 2018; 176: 1–14. doi: 10.1016/j.cognition.2018.02.024 29529396

[45] Spychalska M, Kontinen J, Noveck I, Reimer L, Werning M. When numbers are not exact: Ambiguity and prediction in the processing of sentences with bare numerals. J Exp Psychol: Learn Mem Cogn. 2019; 45(7): 1177. doi: 10.1037/xlm0000644 Epub 2018 Oct 22. 30346210

[46] Shetreet E, Chierchia G, Gaab N. When some is not *Every*: Dissociating scalar implicature generation and mismatch. Hum Brain Mapp. 2014; 35(4): 1503–1514. doi: 10.1002/hbm.22269 23568365 PMC6869420

[47] Spector B. Bare numerals and scalar implicatures. Lang Linguist Compass. 2013; 7(5): 273–294. doi: 10.1111/lnc3.12018

[48] Breheny R. A new look at the semantics and pragmatics of numerically quantified noun phrases. J Semant. 2008; 25(2): 93–139. doi: 10.1093/jos/ffm016

[49] Carston R. Informativeness, relevance and scalar implicature. In: Carston R, Uchida S, editors. Relevance Theory: Applications and Implications. Amsterdam: John Benjamins; 1998. p. 179–236. 10.1075/pbns.37.11car

[50] Chrisomalis S. Numerical notation: A comparative history. Cambridge University Press; 2010, 496 p. 10.1017/CBO9780511676062

[51] Chrisomalis S. Reckonings. MIT Press; 2021. 264 p.

[52] Everett C. Numbers and the Making of Us. Harvard University Press; 2017. 312 p.

[53] Kadosh RC, Henik A, Rubinsten O. Are Arabic and verbal numbers processed in different ways? J Exp Psychol: Learn Mem Cogn. 2008; 34(6): 1377. doi: 10.1037/a0013413 18980402

[54] Proverbio AM, Bianco M, De Benedetto F. Distinct neural mechanisms for reading Arabic vs. verbal numbers: An ERP study. Eur. J Neurosci. 2020; 52(11): 4480–4489. doi: 10.1111/ejn.13938 29753306

[55] Kochari A, Schriefers H. Processing symbolic magnitude information conveyed by number words and by scalar adjectives. Psyxarxiv; 2020. 10.1177/17470218211031158 PMC8793294 34169765

[56] Aronoff M. Competition and the lexicon. In Livelli di Analisi e fenomeni di interfaccia. Atti del XLVII congresso internazionale della società di linguistica Italiana. Roma: Bulzoni Editore 2016 (pp. 39–52)

[57] Gause G.F., The struggle for existence. Williams and Wilkins, Baltimore. 1934.

[58] Roberts G, Fedzechkina M. Social biases modulate the loss of redundant forms in the cultural evolution of language. Cognition. 2018 Feb 1;171:194–201. doi: 10.1016/j.cognition.2017.11.005 29182959

[59] New B, Pallier C, Brysbaert M, Ferrand L. Lexique 2: A new French lexical database. Behav Res Meth Instrum Comput. 2004; 36(3): 516–524. doi: 10.3758/bf03195598 15641440

[60] Peirce J, Gray JR, Simpson S, MacAskill M, Höchenberger R, Sogo H, et al. PsychoPy2: Experiments in behavior made easy. Behav Res methods. 2019; 51(1): 195–203. doi: 10.3758/s13428-018-01193-y 30734206 PMC6420413

[61] Jaeger T. F. (2008). Categorical data analysis: away from ANOVAs (transformation or not) and towards logit mixed models. J Mem Lang, 2008; 59(4), 434–446. doi: 10.1016/j.jml.2007.11.007 19884961 PMC2613284

[62] R Core Team (2013). R: A language and environment for statistical computing. R Foundation for Statistical Computing, Vienna. http://www.R-project.org/

[63] Bates D, Maechler M, Bolker B, Walker S. lme4: Linear Mixed-Effects Models Using Eigen and S4. R package version 1.1–10, 2015; URL http://CRAN.R-project.org/package=lme4.

[64] Tremblay A, Ransijn J. LMERConvenienceFunctions: A suite of functions to back-fit fixed effects and forward-fit random effects, as well as other miscellaneous functions. R package version. 2013; 2: 919–931. https://cran.r-project.org/web/packages/LMERConvenienceFunctions/LMERConvenienceFunctions.pdf

[65] Fox J, Weisberg S. An R Companion to Applied Regression. SAGE: Los Angeles, CA; 2011, 449 p.

[66] Lenth R, Singmann H, Love J, Buerkner P, Herve M. Emmeans: Estimated marginal means, aka least-squares means. R package version. 2018; 1(1): 3. https://github.com/rvlenth/emmeans

[67] Damian MF. Asymmetries in the processing of Arabic digits and number words. Memory & Cognition. 2004 Jan;32(1):164–71. doi: 10.3758/bf03195829 15078053

[68] Ferrand L. Why naming takes longer than reading? The special case of Arabic numbers. Acta Psychologica. 1999 Jan 1;100(3):253–66.

[69] Marty P, Chemla E, Spector B. Interpreting numerals and scalar items under memory load. Lingua. 2013; 133: 152–163. 10.1016/j.lingua.2013.03.006

