# Correction: When *eleven* does not equal *11*: Investigating exactness at a number’s upper bound

**DOI:** 10.1371/journal.pone.0303710

**Published:** 2024-05-09

**Authors:** Ira Noveck, Martial Foegel, Kira Van Voorhees, Giuseppina Turco

The second author’s name is spelled incorrectly. The correct name is: Martial Foegel. The correct citation is: Noveck I, Foegel M, Van Voorhees K, Turco G (2022) When eleven does not equal 11: Investigating exactness at a number’s upper bound. PLoS ONE 17(4): e0266920. https://doi.org/10.1371/journal.pone.0266920.
